# Evaluation of a Screening System for Obesogenic Compounds: Screening of Endocrine Disrupting Compounds and Evaluation of the PPAR Dependency of the Effect 

**DOI:** 10.1371/journal.pone.0077481

**Published:** 2013-10-14

**Authors:** Anna Pereira-Fernandes, Heidi Demaegdt, Karine Vandermeiren, Tine L. M. Hectors, Philippe G. Jorens, Ronny Blust, Caroline Vanparys

**Affiliations:** 1 Systemic Physiological and Ecotoxicological Research (SPHERE), Department of Biology, University of Antwerp, Antwerp, Belgium; 2 Chemical Safety of the Food Chain, Veterinary and Agrochemical Research Centre (CODA-CERVA), Brussels, Belgium; 3 Department of Clinical Pharmacology/Clinical Toxicology, University of Antwerp, Antwerp, Belgium; Clermont Université, France

## Abstract

Recently the environmental obesogen hypothesis has been formulated, proposing a role for endocrine disrupting compounds (EDCs) in the development of obesity. To evaluate this hypothesis, a screening system for obesogenic compounds is urgently needed. In this study, we suggest a standardised protocol for obesogen screening based on the 3T3-L1 cell line, a well-characterised adipogenesis model, and direct fluorescent measurement using Nile red lipid staining technique. In a first phase, we characterised the assay using the acknowledged obesogens rosiglitazone and tributyltin. Based on the obtained dose-response curves for these model compounds, a lipid accumulation threshold value was calculated to ensure the biological relevance and reliability of statistically significant effects. This threshold based method was combined with the well described strictly standardized mean difference (SSMD) method for classification of non-, weak- or strong obesogenic compounds. In the next step, a range of EDCs, used in personal and household care products (parabens, musks, phthalates and alkylphenol compounds), were tested to further evaluate the obesogenicity screening assay for its discriminative power and sensitivity. Additionally, the peroxisome proliferator activated receptor γ (PPARγ) dependency of the positive compounds was evaluated using PPARγ activation and antagonist experiments. Our results showed the adipogenic potential of all tested parabens, several musks and phthalate compounds and bisphenol A (BPA). PPARγ activation was associated with adipogenesis for parabens, phthalates and BPA, however not required for obesogenic effects induced by Tonalide, indicating the role of other obesogenic mechanisms for this compound.

## Introduction

Recently, The Endocrine Society redefined endocrine disrupting compounds (EDCs) as exogenous chemicals, or mixtures of chemicals that interfere with any aspect of hormone action [1]. Different international research programs have been developed to evaluate the health impact of EDC exposure (e.g. US-EPA: Endocrine disruptor screening program (EDSP), OECD: Endocrine Disruptor Testing and Assessment Task Force (EDTA)). In these programs general effects of EDCs on reproductive and developmental toxicity or carcinogenicity are evaluated. However, recently EDCs have been pointed out as first line candidates for possible effects on other endocrine organs (e.g. adipose tissue, pancreas,…) related to the development of metabolic diseases such as obesity or diabetes, broadening the term of endocrine disruption to ‘metabolic disruption’ [2,3]. The elaborate study of Grun et al. [4] showing the adipogenic potential of tributyltin (TBT) after *in vitro* and *in utero* exposure, resulted in the formulation of the obesogen hypothesis, postulating that exposure to environmental pollutants early in life or throughout life has an impact on obesity development. 

Peroxisome proliferator-activated receptor γ (PPARγ) is a nuclear receptor, acting as regulator for adipocyte differentiation and lipid metabolism [2]. Several authors suggested the potential direct link between PPARγ agonists and obesogens [5,6]. Indeed, recently Taxvig et al. [6] showed that PPARγ agonists frequently induce adipogenesis. However, PPARγ receptor activation is neither a requirement nor an assurance for adipogenesis, indicating the importance of other mechanisms of action such as for example glucocorticoid receptor activation [7]. Therefore, a combination of the 3T3-L1 adipogenesis assay with a PPARγ activation assay will be necessary as a first line screening system for obesogens, as is suggested by the OECD [8]. The development of these standardised, reproducible *in vitro* screening techniques will be essential for a first identification of potential obesogenic compounds which can then be further tested in a second step using *in vivo* assays. 

EDCs used in personal and household care products are of great human interest due to daily, multiple application or long time exposure. The endocrine disrupting effects of parabens, phthalates, alkylphenols and musks have already been studied *in vitro* or *in vivo* [9-12]. However, the obesogenic effects of those compounds are scarcely documented, despite the fact that some compounds such as musks are highly lipophilic (Log k_ow_ = ± 5.00), and have been detected in human adipose tissue at average concentrations up to 361 and 132 ng/g lipid weight for Galaxolide and Tonalide (TON) respectively [13-17]. 

Although 3T3-L1 cells are already widely used for study of adipogenesis, they are only recently used for screening of environmental obesogens and never thoroughly evaluated for that purpose. Moreover, the direct fluorescence measurement of Nile red stained cells for quantification of adipocyte differentiation is only scarcely used in literature, despite the study of Aldridge et al. [18] that indicated that this method is the most quantitative and least subjective method for measuring adipogenesis. Therefore, the aim of this study is to develop a reproducible, standardised protocol for the adipocyte differentiation assay to use as *in vitro* tool for environmental obesogen screening, based on this promising fluorescence-based quantification [18]. This adipocyte differentiation assay was further evaluated by screening different known and unknown environmental obesogens used in personal and household care products. Since PPARγ signalling is known to be one of the major regulators of adipocyte differentiation, PPARγ transactivation and PPARγ antagonist studies were additionally conducted to evaluate its role in the obesogenic effect of the tested compounds. 

## Materials and Methods

### Chemicals

Cell culture reagents were obtained from Life Technologies (Merelbeke, Belgium) unless otherwise indicated. Alkylphenol compounds, parabens and Ethylene brassylate were obtained from Sigma-Aldrich. Musk xylene was purchased from Penta Manufacturing Company (Livingstone, USA). Phthalates were obtained from Campro Scientific (Veenendaal, Nederland). TBT-Cl was purchased from Acros Chemicals (Geel, Belgium). Rosiglitazone and T0070907 were obtained from Cayman Chemicals (MI, USA). Stock concentrations of test compounds were made in 100% DMSO (Sigma-Aldrich). For 3T3-L1 experiments stock concentrations were a thousand times diluted in media to reach a final maximal concentration of 0.1% DMSO, whereas for CALUX experiments stock concentrations were hundred times diluted in media to reach 1% DMSO as final concentration. Non-cytotoxic concentrations were used for analysis, as determined in the AlamarBlue viability assay [19]. 

### 3T3-L1 routine cell culture

3T3-L1 mouse pre-adipocyte cells (American Type Culture Collection CRL-173®, LGC Promochem GmbH, Wesel, Germany) were maintained in T75 culture flasks (Nunc, VWR, Leuven, Belgium) in growth medium composed of high glucose (4.5 g/L) Dulbecco’s modified Eagle’s medium (DMEM) supplemented with 10% (v/v) Heat Inactivated Newborn Calf serum, 100 U/mL Penicillin, 100 µg/mL Streptomycin and 1 mM sodium pyruvate (Sigma-Aldrich, Bornem, Belgium). All cell culture experiments were performed in a 5% CO_2_ atmosphere at 37 °C. Before reaching confluence, cells were detached using 0.25% (v/v) Trypsin/EDTA. Cells were used at passage 6-11.

### Experimental setup: 3T3-L1 lipid accumulation test

**Figure 1:**
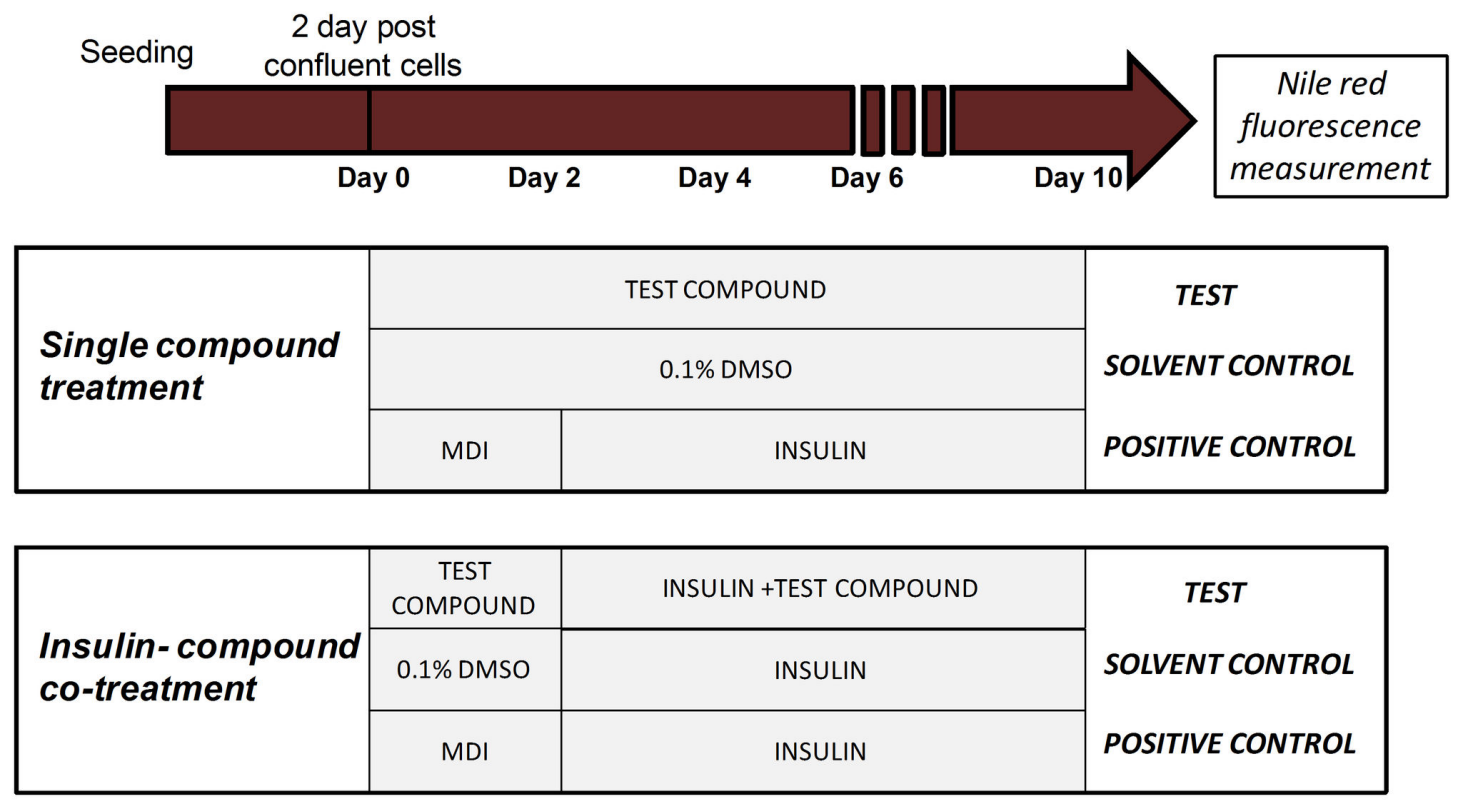
Overview of the experimental setup of one compound and insulin-compound co-treatment experiment of the 3T3-L1 cells. Medium was changed every 2-3 days. *Insulin: 10 µg/mL* *insulin; MDI: 10 µg/µL* *insulin, 0.25 µM* *dexamethasone and 0.5* *mM* *isobutylmethylxanthin*.

Cells were seeded in 24-well plates at a density of 50,000 cells/well and grown until confluent. At the second day post confluence, denoted as day 0, growth medium was replaced by exposure medium containing 10% Heat Inactivated Foetal Bovine Serum (FBS) instead of Newborn Calf Serum and supplemented with test compounds. Two exposure scenarios were tested for each test compound: a single-compound treatment and an insulin-compound co-treatment (Figure 1). Concerning the *single-compound treatment*, cells were exposed from day 0 until day 10, refreshing the medium with added compound every 2-3 days. A solvent control (0.1% DMSO) was included in each experiment. Regarding the *insulin supplemented exposure*, cells were first exposed to the test compound alone from day 0 until day 2. Thereafter, medium was changed to medium with the test compound and insulin (10 µg/mL) and refreshed every 2-3 days until day 10. A solvent control was included in each experiment, consisting of 0.1% DMSO from day 0-2 and 0.1% DMSO + insulin (10 µg/mL) from day 2-10. 

For both exposure scenarios, a positive control was always included to control the differentiation ability of the cells. As a positive control, cells were stimulated with MDI hormonal cocktail (0.5 mM isobutylmethylxanthin, 0.25 µM dexamethasone and 10 µg/mL insulin) from day 0-2 and thereafter with only insulin (10 µg/mL) for another 8 days. As for the test compounds, the medium of the positive control was changed every 2-3 days. 

Finally, as a mechanistic control, each positive test compound was co-exposed with the PPARγ-antagonist T0070907 to evaluate the receptor mediated mechanism of lipid accumulation. In this set-up, T0070907 was added to the single-compound treatment during 10 days and medium was refreshed every 12h to counteract T0070907 breakdown [20]. In a primary experiment, a concentration range of T0070907 (0.3-10 µM) was tested with the reference compounds, rosiglitazone and TBT, to select a maximal active concentration (10 µM). This concentration was then further used to evaluate the lipid accumulation mechanism of the positive test compounds. 

 For all exposure scenario’s, cells were finally stained with Nile red and Oil Red O at day 10 for intracellular lipid measurements. 

### Intracellular lipid measurement and visualisation

At day 10, the intracellular lipid content was quantified using the commercially available Nile red stain (AdipoRed assay Reagent; Lonza, Walkersville, MD) following the manufacturer’s instructions. The solution of the hydrophilic stain Nile red, is a reagent that becomes fluorescent when it is partitioned in a lipophilic environment, with a specific emission maximum in lipid droplets, making the direct fluorescent measurement an accurate, fast technique for quantification of lipid accumulation. Additionally, fluorescent pictures were then taken to visualise the lipid droplets using a JuLi^TM^ smart fluorescent cell analyser (International Medical Products, Brussels, Belgium). Furthermore, light microscopic photographs were taken of Oil Red O stained cells as previously described in [21].

### Gene expression analysis using Real time PCR

Gene expression analysis was performed as described in Pereira-Fernandes et al. [22]. Briefly, RNA extraction and cDNA reverse transcription was performed using respectively RNeasy kit form Qiagen (Antwerp, Belgium) and Revert Aid TM *H Minus* First strand cDNA synthesis kit for RT-PCR (Thermo Fisher Scientific, Zellik, Belgium) according to manufacturer’s instructions. Highly purified salt-free ‘Oligogold’ primers (Eurogentec, Seraing, Belgium) were selected for the target gene adipocyte specific protein 2 (*aP2*; NM_024406) (FW: AGT-GGA-AAC-TTC-GAT-GAT-TAC-ATG-ATG-AA; RE: GCC-TGC-CAC-TTT-CCT-TGT-G) and household gene TATA binding protein (Tbp; NM_013684) (FW: ACC-CTT-CAC-CAA-TGA-CTC-CTA-TG; RE: ATG-ATG-ACG-GCA-GCA-AAT-CGC) [23,24]. Real-time PCR reaction master mix was used following the manufacturer’s instructions (Brilliant^®^ II SYBR^®^ Green QPCR mastermix, Agilent Technologies, Santa Clara, CA). According to the equation of Pfaffl [25] the expression values of the target gene (*aP2*) were normalized by comparison to the household gene (*Tbp*), and an exposure versus solvent control relative expression ratio was calculated. 

### PPARγ Chemically Activated LUciferase eXpression (CALUX) assay

In the PPARγ CALUX^®^ cell line (developed by BioDetection Systems (Amsterdam)), U-2 OS human osteoblast cells have been stably transfected with a vector construct containing firefly luciferase cDNA under the control of a promoter containing peroxisome proliferator hormone response elements (PPREs). Furthermore, these cells have been stably co-transfected with a vector construct containing the cDNA of human PPARγ (transcript variant 2) under control of the SV40 promoter. The addition of PPARγ agonists to the cell medium results in expression of the luciferase enzyme. Hence this is a quantifiable cell based assay for PPARγ agonists. The cells also contain an antibiotic resistance gene, pSV_2_ neo which was introduced into the cells for efficient selection of the expressing cells. 

The cells were cultured in T75 flasks (Nunc) in DMEM/F12 medium supplemented with 10 U/mL penicillin and 10 µg/mL streptomycin; 1% (v/v) of a stock solution of non essential amino acids; and 7,5% (v/v) FBS (all from Fischer Scientific Belgium). The cells were grown in 5% CO_2_ at 37°C until confluent.

For the experiments, cells were seeded in DMEM/F12 (also supplemented, but 7,5 % (v/v) FBS is replaced with 5 % (v/v) serum stripped with dextran coated charcoal (DCC-FCS; BDS, Amsterdam)) at a concentration of 80,000 cells/well in 96-wells plates and incubated at 37°C and 5% CO_2_ for 24h. For the following 24 hours, cells were incubated in the presence of a concentration range of the relevant compounds. Then, cells were lysed and luciferase activity was measured in a luminometer (Berthold Tristar) by adding glowmix (BDS, Amsterdam). 

## Results

### Evaluation of the 3T3-L1 adipocyte differentiation assay: reference compounds

 To evaluate the stability and reproducibility of the adipocyte differentiation assay (using direct fluorescence measurement of Nile red) for *in vitro* obesogenic screening, two well-known adipogenic compounds acting through PPARγ activation, Rosiglitazone (ROSI) and Tributyltin (TBT), were selected. For standardization of the procedure the degree of lipid accumulation (DLA) was calculated as the relative fluorescent units (RFU) of the test compound condition relative to the RFU of the solvent control (0.1% DMSO). Different quality criteria were introduced to evaluate the assay performance. 

First, the sensitivity of the assay was guaranteed by setting minimum levels for the DLA of the positive control MDI, only experiments with a DLA of MDI greater than 10 were used. This means that only cells with a 10 times higher lipid content of the positive control compared to the solvent control are considered sensitive. Including experiments with MDI values with lower DLA would restrain the dynamic range of the measurements and therefore hinder the detection of weak obesogenic compounds. 

Secondly, to assure the biological meaning of statistically significant effects, a lipid accumulation threshold (LAT) is proposed, based on the limit of quantification (LOQ) used in analytical chemistry techniques. LOQ is defined as the lowest amount of a chemical that can be quantitatively determined with suitable precision and accuracy and is calculated as the blank value + 10*SD [26]. In this way, the LAT is based on the variation of the solvent control and calculated as the solvent control value (1) + 10*SD of the solvent control over all experiments performed for this paper. Two different exposure scenarios were evaluated for this assay: single-compound treatment and insulin-compound co-treatment. For the single-compound treatment, a LAT of 1.76 was calculated. 

In the insulin-compound co-treatment experiments, the combination of a test compound together with insulin exposure was tested. In that way, chemicals that are unable to induce differentiation on their own, but need insulin for conversion to mature adipocytes can also be detected. In these experiments, insulin is the solvent control, resulting in the degree of lipid accumulation compared to insulin (DLAI). A DLAI of 4 was required for the positive control MDI, ensuring the sensitivity of the assay and a broad dynamic range for detection of weaker compounds. The LAT value for these experiments was based on the variation of the insulin treated cells (as solvent control) and corresponds to 2.17.

Using the LAT-based method, compounds inducing a statistically significant DLA/DLAI in at least 2 subsequent concentrations compared to the solvent control, but lower than the LAT are considered weak obesogens, whereas compounds inducing a significant DLA/DLAI higher than the LAT are considered strong obesogens. For these obesogens the lowest concentration causing a significant increase in DLA or DLAI compared to their respective solvent control (LOEC) was determined. This new method for selection of obesogenic compounds (LAT-based method) was compared to the strictly standardized mean difference (SSMD) method calculated using the method-of-moment method as proposed by Zhang [27,28] for selection of hits in high throughput screening assays. Considering *X* and *X*_*sc*_ as the mean of respectively the test substance and the solvent control, and *s* and *s*_*sc*_ the standard deviation of the test sample and the solvent control respectively, the SSDM value can be calculated as: SSMD= (X-X_SC_) / √(s^2^+s_SC_^2^).

Compounds inducing a SSMD value higher than 4.7 are considered strong obesogens, whereas compounds inducing SSMD value between 2 and 4.7 are selected as weak obesogens based on the extended 1-2-3 rule described in Zhang [27]. Compounds causing a SSMD lower than 2 are considered non-obesogenic.

In a precautionary approach, we suggest combining both LAT and SSMD selection methods. In this paper, the strongest obesogenic effect defined by either one of these methods was considered to classify the compounds. 

**Figure 2:**
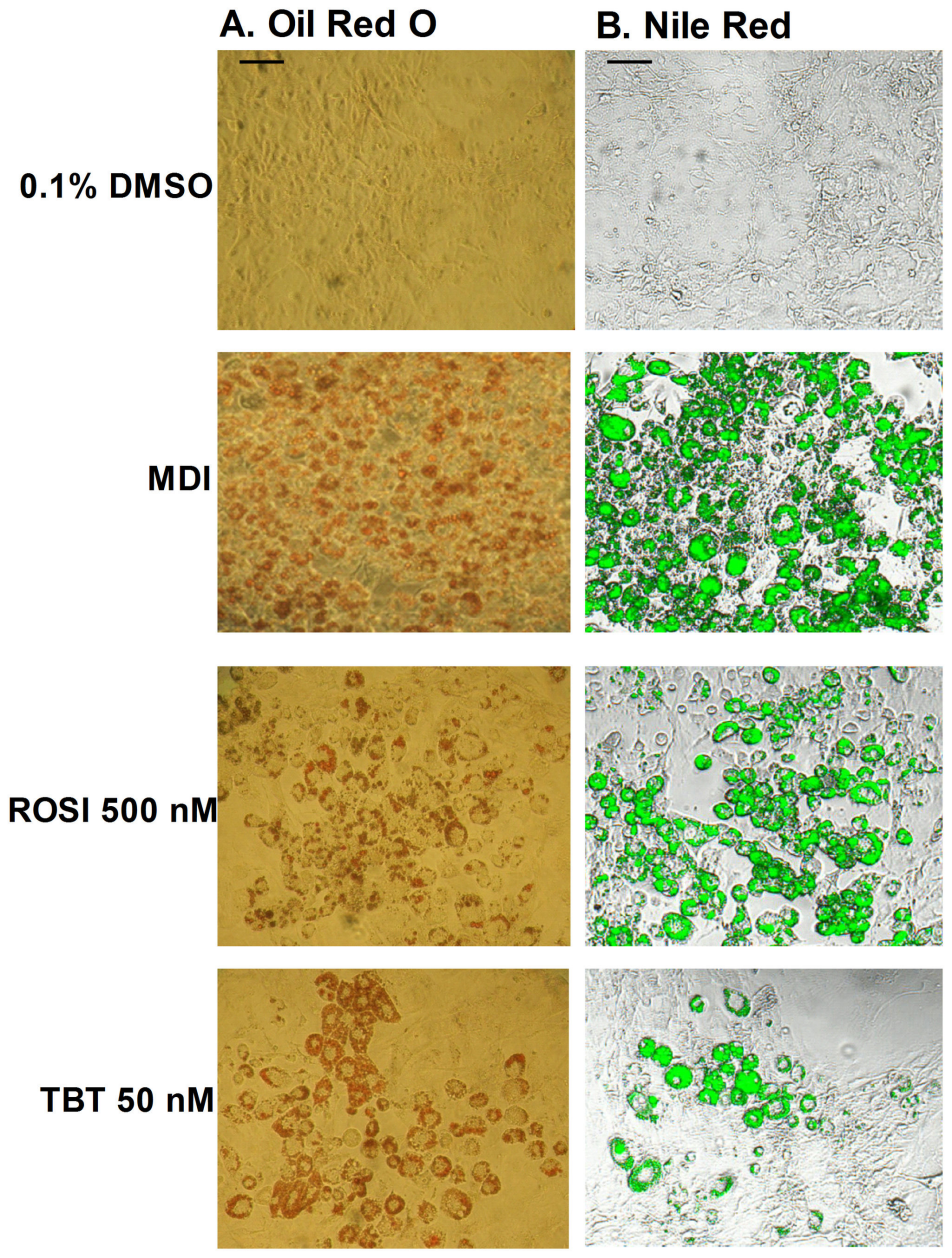
Pictures of Oil Red O and Nile Red stained 3T3-L1 cells after 10 days of exposure to reference compounds and solvent/positive control. Light microscopic pictures (A; Oil Red O staining) and fluorescent (b; Nile Red staining) were taken. Pictures were only taken for visualisation of the differentiation and not for quantification. Scale bar represents 50µm.

**Figure 3:**
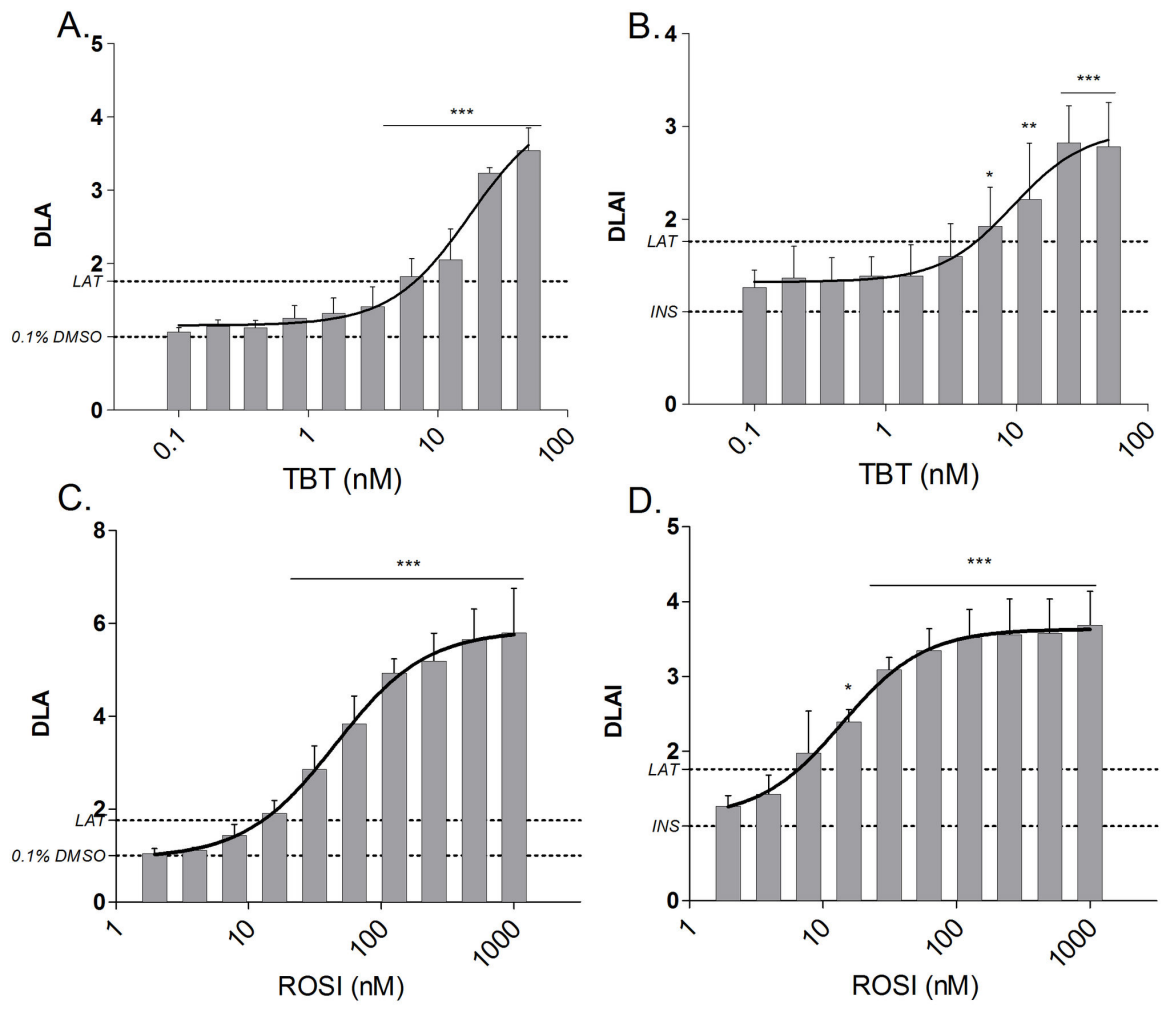
Effect of reference compounds on adipocyte differentiation in 3T3-L1 cells after 10 days of exposure. Fluorimetric quantification of lipid accumulation was performed using Nile Red staining. The degree of lipid accumulation induced by TBT in absence (A.) or presence (B.) of insulin and by ROSI in absence (C.) or presence (D.) of insulin are represented as mean (±SD) of three independent experiments Data represent mean (±SD) of the degree of lipid accumulation (with insulin) (DLA(I)) of three independent experiments (each with 4 replicates) (n=3). Significant differences with solvent control (0.1% DMSO or INS) are indicated with asterisks (One Way ANOVA Dunnet’s post hoc test; *p≤0.05; **p≤0.01; ***p≤0.001). Additionally, a sigmoidal dose−response curve was fitted (GraphPad Prism). *LAT: Lipid* *Accumulation* *Threshold*.

The reference compounds (ROSI and TBT) and the positive control (MDI) induced the differentiation of the 3T3-L1 cells (Figure 2). Moreover, the reference compounds TBT and ROSI induced the differentiation of 3T3-L1 cells dose-dependently, with or without insulin (Figure 3). The maximal degree of lipid accumulation (DLA(I)_MAX_) was higher for ROSI compared to TBT. Concerning TBT, the LOEC values were the same in presence or absence of insulin, whereas for ROSI the LOEC was lower in insulin-compound co-treatment experiments (Table 1). Both compounds were active in the nM range. The coefficient of variation calculated on the DLA_max_ value was 8.51% for TBT and 16.51% for ROSI. Both single-compound and insulin-compound co-treatment experiments revealed TBT and ROSI as strong obesogens. 

**Table 1:** Summary of the obesogenic effects of reference and test compounds using the 3T3-L1 lipid accumulation assay.

***COMPOUND***	***CAS-NR***		***CONC. RANGE TESTED ½ dilution***	***SINGLE COMPOUND TREATMENT***					***INSULIN - COMPOUND CO-TREATMENT***					***PPARγ CALUX***				
				*Effect*		*DLA_max_*	*[DLA_max_]*	*LOEC*	*Effect*		*DLAI_max_*	*[DLAI_max_]*	*LOEC*	*Effect*		*FI_max_*	*[FI_max_]*	*LOEC*
				*LAT*	*SSMD*				*LAT*	*SSMD*				*LAT*	*SSMD*			
**REFERENCE COMPOUNDS**																		
Rosiglitazone	122320-73-4	ROSI	*1.95-1000nM*	**S**	**S**	5.8±0.96	1 µM	31 nM	**S**	**S**	3.69±0.44	1 µM	16 nM	**S**	**S**	17,25±3.06	10 µM	30 nM
Tributyltin	1461-22-9	TBT	*0.10-50nM*	**S**	**S**	3.55±0.30	50 nM	6.25 nM	**S**	**W**	2.78±0.47	50 nM	6.25 nM	**S**	**S**	3,09±0.37	30 nM	3 nM
**ALKYLPHENOL COMPOUNDS**																		
4-Nonylphenol	104-40-5	NP	*0.05-25µM*	**ANTI?**		/	/	/	**ANTI?**		/	/	/			/	/	/
4-Octylphenol	1806-26-4	OP	*0.05-25µM*			/	/	/			/	/	/			/	/	/
Bisphenol A	80-05-7	BPA	*0.10-50µM*	**W**	**W**	1.38±0.09	50 µM	12.5 µM	**W**	**W**	1.85±0.21	50 µM	25 µM	**W**		1.22±0.1	10 µM	1 µM
**PHTHALATES**																		
Butyl benzyl phthalate	85-68-7	BBP	*0.10-50µM*	**S**	**S**	1.85± 0.11	50 µM	50 µM	**S**	**S**	2.59±0.27	50 µM	25 µM	**S**	**S**	2.08±0.14	10 µM	1 µM
Di-n-butylphthalate	84-74-2	DBP	*0.10-50µM*			/	/	/	**W**	**S**	1.91±0.15	50 µM	12.5 µM	**W**		1.50±0.30	50 µM	10 µM
Bis (2-ethylhexyl)phthalate	117-81-7	DEHP	*0.10-50µM*			/	/	/			/	/	/	**W**	**W**	1.28±0.07	10 µM	3 µM
Di-iso-butyl phthalate	84-69-5	DiBP	*0.10-50µM*	**W**	**W**	1.43± 0.13	50 µM	25 µM	**S**	**S**	2.68±0.26	50 µM	12.5 µM	**W**	**W**	1.35±0.13	10 µM	10 µM
Di-iso-nonyl phthalate	28553-12-0	DiNP	*0.10-50µM*			/	/	/			/	/	/			/	/	/
**PARABENS**																		
Methylparaben	99-76-3	MP	*0.39-200µM*			/	/	/	**W**	**W**	1.97±0.20	200 µM	100 µM	**W**	**W**	1.35±0.08	100 µM	30 µM
Ethylparaben	120-47-8	EP	*0.39-200µM*			/	/	/	**W**	**S**	2.10±0.18	200 µM	100 µM	**W**	**W**	1.71±0.17	100 µM	30 µM
Propylparaben	94-13-3	PP	*0.39-200µM*	**S**	**W**	1.91±0.42	200 µM	100 µM	**S**	**W**	2.33±0.41	200 µM	100 µM	**W**	**S**	1.75±0.09	100 µM	10 µM
Butylparaben	94-26-8	BP	*0.19-100µM*	**S**	**W**	3.20±0.77	100 µM	50 µM	**S**	**W**	2.40±0.46	100 µM	50 µM	**S**	**W**	2.01±0.19	100 µM	10 µM
**MUSKS**																		
Musk xylene	81-15-2	MX	*0.10-50µM*	**W**	**W**	1.65±0.24	50 µM	6.25 µM	**W**	**S**	1.69±0.09	50 µM	25 µM			/	/	/
Tonalide	1506-02-1	TON	*0.06-30µM*	**S**	**W**	2.02±0.33	30 µM	7.5 µM	**W**	**W**	2.11±0.23	15 µM	0.94 µM			/	/	/
Ethylene Brassylate	105-95-3	EB	*0.19-100µM*			/	/	/			/	/	/			/	/	/

Values are represented as mean of three experiments performed with 4 replicates (±SD). Weak (W) and strong (S) effects, are calculated based on the lipid accumulation threshold (LAT) and the strictly standardized mean difference (SSMD) methods. Compounds that were not effective, are represented with a blank case. In this paper, the strongest obesogenic effect defined by either one of these methods was considered to classify the compounds. *DLA(I*)_*max*_ *: maximal degree of lipid accumulation (with insulin*)*; FI*_*max*_ *= maximal fold induction of PPARγ activation compared to solvent control; [X*]*: concentration inducing X; LOEC: Lowest Observed Effect Concentration inducing a significant DLA(I*)* or FI compared to the solvent control; ANTI?: Potentially inhibiting effect.*

### Screening of compounds: Single compound treatment

**Figure 4:**
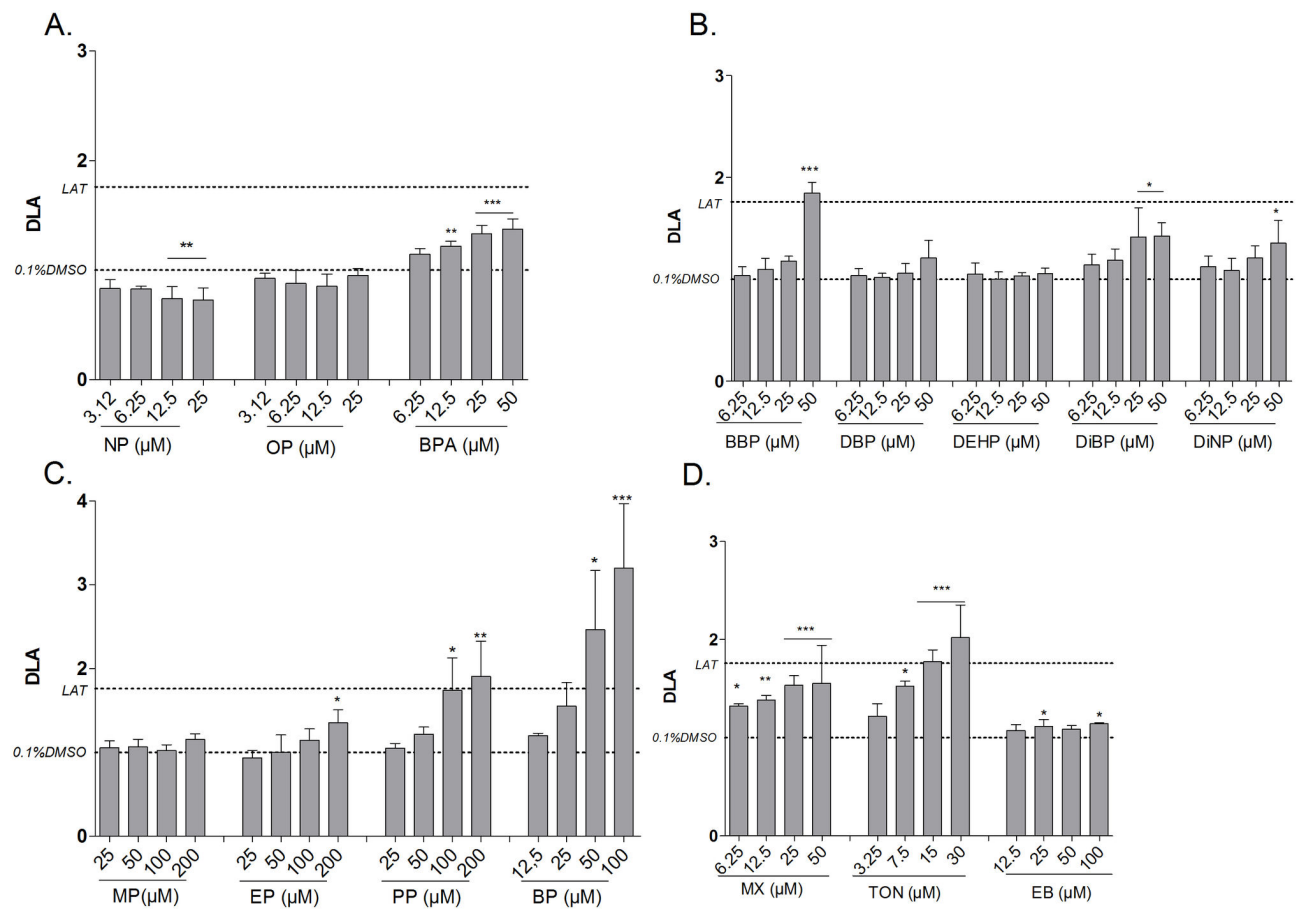
Effect of test compounds on adipocyte differentiation in 3T3-L1 cells after 10 days of exposure. Fluorimetric quantification of lipid accumulation was performed using Nile Red staining. The degree of lipid accumulation (DLA) induced by Alkylphenol (A.), Phthalate (B.) Paraben (C.) and Musk compound (D.) exposure are represented as mean (±SD) of three independent experiments (each with 4 replicates) (n=3). Significant differences with solvent control (0.1% DMSO) are indicated with asterisks (One Way ANOVA Dunnet’s post hoc test; *p≤0.05; **p≤0.01; ***p≤0.001). *LAT: Lipid* *accumulation* *threshold* .

To further evaluate the screening potential of the assay, a diverse set of chemicals used in personal and household care products were tested for potential effects on differentiation (Table 1, Figure 4). For all chemicals a broad concentration range (10 concentrations) was tested, however for clarity of the figure only the four highest effect concentrations are shown on Figure 4. Concerning the alkylphenol compounds, Bisphenol A (BPA) was screened as a weak obesogen causing a DLA_max_ of 1.38 with a LOEC of 12.5 µM, whereas Nonylphenol (NP) suppressed the differentiation with a DLA of 0.72 (Table 1, Figure 4A). Regarding the phthalates butylbenzyl phthalate (BBP) was the only strong obesogen (DLA_max_= 1.85), and 50 µM concentration was needed to induce an effect. Di-iso-butyl phthalate (DiBP) acted as a weak obesogen causing a DLA_max_ of 1.43 and was already effective at 25 µM (Table 1, Figure 4B). Butyl- and propylparaben were strong obesogens inducing a DLA_max_ of 1.91 and 3.20 respectively and a LOEC of 100 µM and 50 µM (Table 1, Figure 4C). Considering the musk compounds, only Tonalide (TON) was able to significantly induce the differentiation of adipocytes above the LAT and was therefore selected as a strong obesogen. This effect was dose dependent (Figure 4D), resulting in a DLA_max_ of 2.02 and a LOEC of 4.5 µM (Table 1). Although the effect of Musk Xylene (MX) showed a clear dose response profile, it was classified as a weak obesogen (Figure 4D).

**Figure 5:**
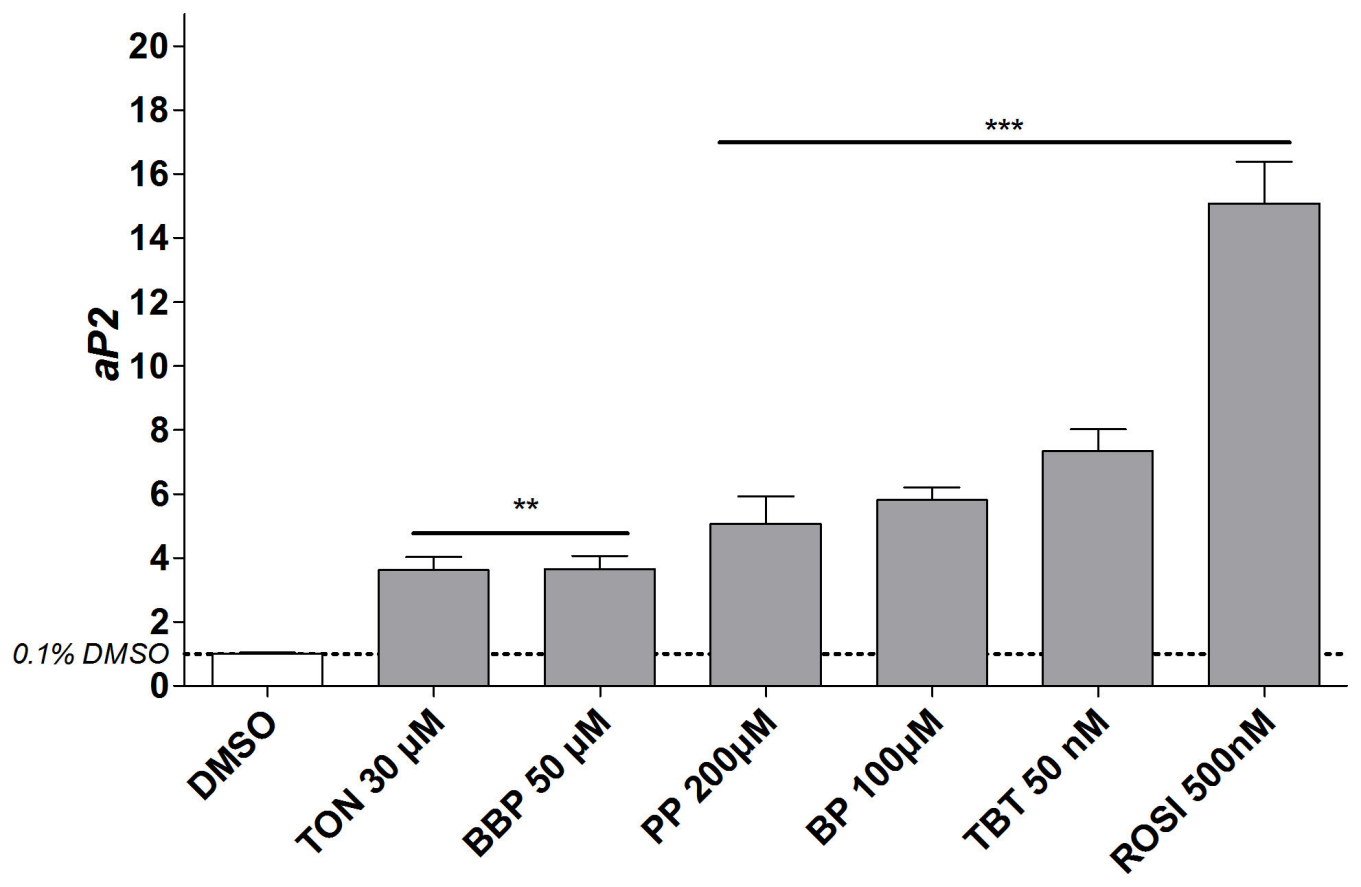
Gene expression of the adipocyte specific gene *adipocyte* *specific* protein *2* (*aP2*) measured by Real-time PCR at day 10. Data are represented as the mean fold change relative to the solvent control of 3 biological replicates (mean±stdev; n = 3). Significant differences between the conditions were analysed with One Way ANOVA (Dunnet’s post hoc test; *p≤0.05; **p≤0.01; ***p≤0.001).

The obesogenic effects of compounds screened as strong obesogens, were further confirmed based on the gene expression level of the adipocyte marker gene aP2 (Figure 5).

**Figure 6:**
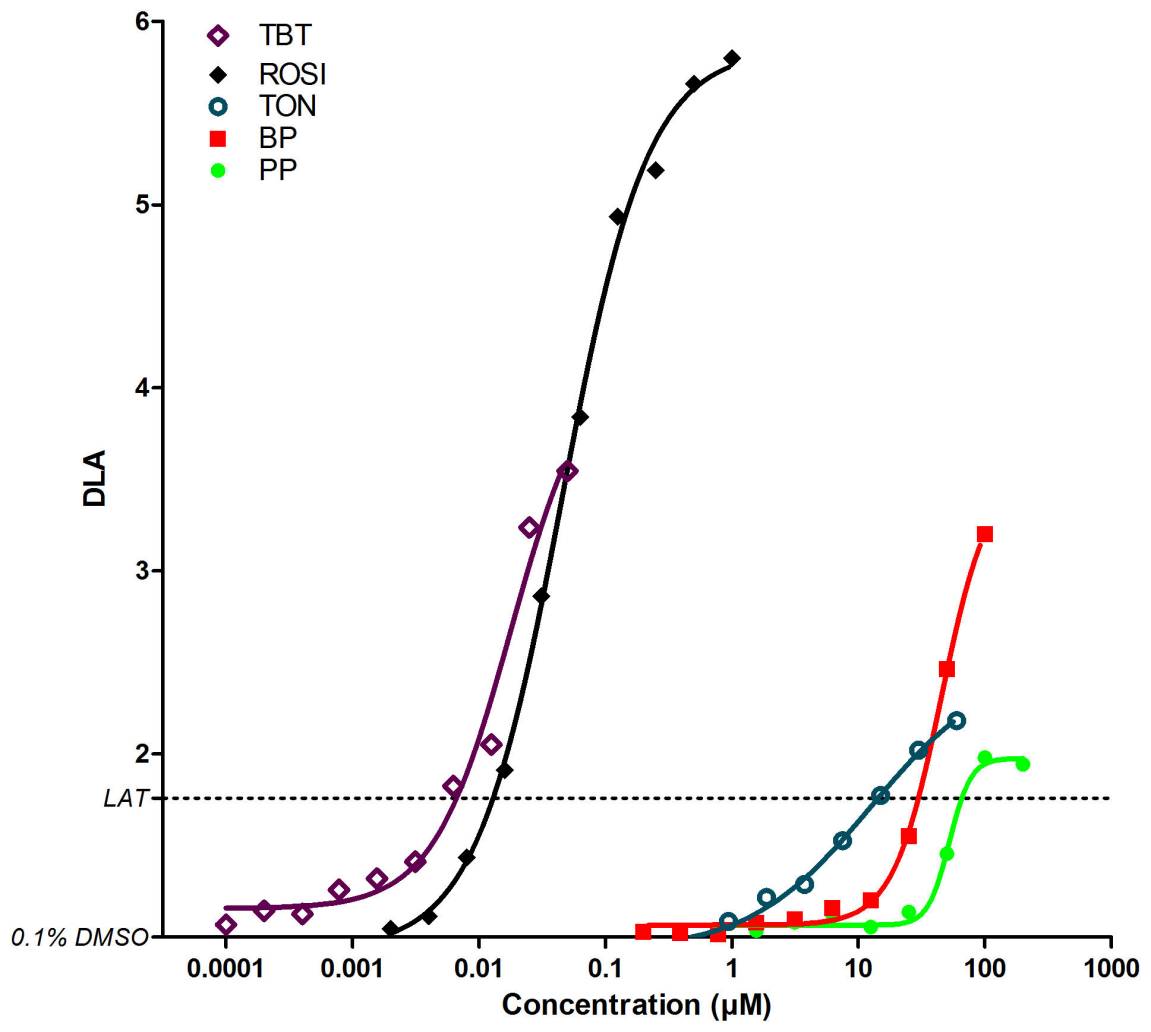
Dose-response relationships of strong obesogens based on adipocyte differentiation in 3T3-L1 cells. Data represent the same data as figure 2 and 3 but with 10 concentrations shown. Mean of the degree of lipid accumulation (DLA) of three independent experiments are shown. *LAT: Lipid* *accumulation* *threshold* .

Overall strong obesogenic compounds, except BBP in the single compound treatment, induced a dose dependent increase in DLA. To compare the potency of those strong obesogens, DLA values at all tested concentrations are visualized in Figure 6 and dose-response curves were constructed. The term EC_LAT_ was introduced, meaning the concentration causing a DLA equal to the LAT. Based on the dose response curves compounds can be ranked using their EC_LAT_ values, from high to low potency this results in TBT (7 nM) > ROSI (13 nM) > TON (14 µM) > BP (30µM) > PP (66µM). 

### Screening of compounds: Insulin-compound co-treatment

**Figure 7:**
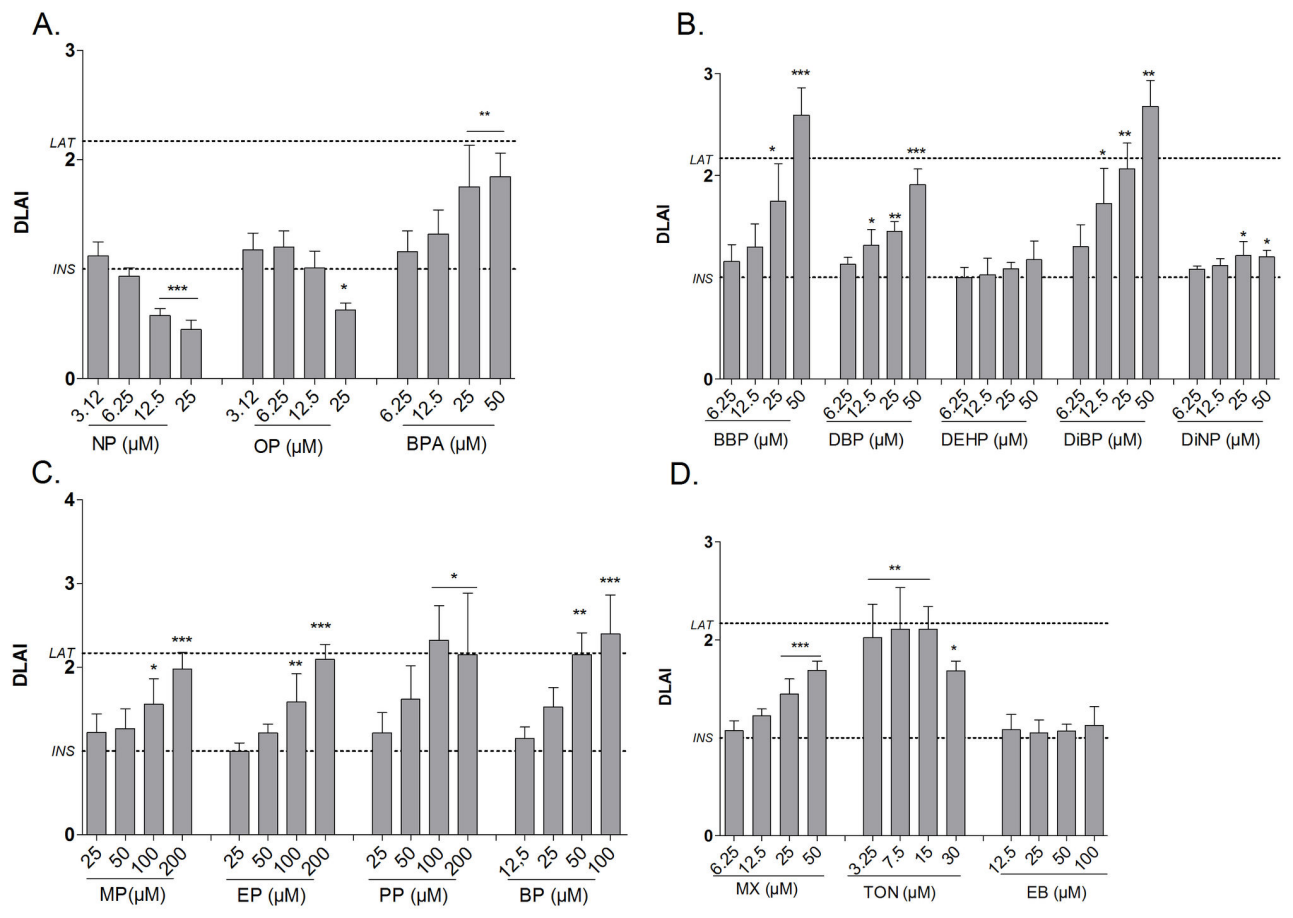
Effect of combined insulin and test compound exposure on adipocyte differentiation in 3T3-L1 cells after 10 days of exposure. Fluorimetric quantification of lipid accumulation was performed using Nile red staining. The degree of lipid accumulation with insulin (DLAI) induced by Alkylphenol (A.), Phthalate (B.) Paraben (C.) and Musk compound (D.) exposure are represented as mean (±SD) of three independent experiments (each with 4 replicates) (n=3). Significant differences with control (INS) are indicated with asterisks (One Way ANOVA Dunnet’s post hoc test; *p≤0.05; **p≤0.01; ***p≤0.001); *DLAI: Degree* *of* *Lipid* *Accumulation* *with* *Insulin; LAT: Lipid* *accumulation* *threshold* .

Besides the effect of these compounds on the initiation of adipocyte differentiation, the evaluation of the potential of compounds to increase the insulin mediated adipogenic effect was also included in this study with the insulin-compound co-treatment (Table 1, Figure 7). The only alkylphenol compound that inhibited the insulin mediated differentiation was NP, by decreasing the DLAI dose dependently starting at 12.5µM. BPA was screened as a weak obesogen with a DLAI_max_ of 1.85 reached at 50µM (Table 1, Figure 7A). The phthalate compounds BBP and DiBP were able to strongly induce the differentiation of adipocytes in a dose dependent manner when insulin was present leading to a DLAI_max_ of 2.59 and 2.68 and LOECs of 25µM and 12.5µM respectively. Di-n-butylphthalate (DBP) was screened as a strong obesogen, in presence of insulin but was effective at the same concentration than previously mentioned phthalates (LOEC=12.5µM) (Table 1, Figure 7B). The paraben compounds EP, PP and BP strongly induced the differentiation of the 3T3-L1 cells with DLAI_max_ of respectively 2.10, 2.33 and 2.40, whereas methylparaben (MP) reacted as a weak obesogen inducing a DLAI of 1.97. All parabens were effective at 100µM and the LOEC of BP was 50µM (Table 1, Figure 7C). Comparable to the one compound treatment, addition of insulin increased the potential of the parabens with increasing alkyl chain. Considering musk compounds, MX and TON were selected as strong and weak obesogens respectively inducing a DLA_MAX_ of 1.69 and 2.11 (Table 1, Figure 7D). 

### PPARγ antagonist tests

**Figure 8:**
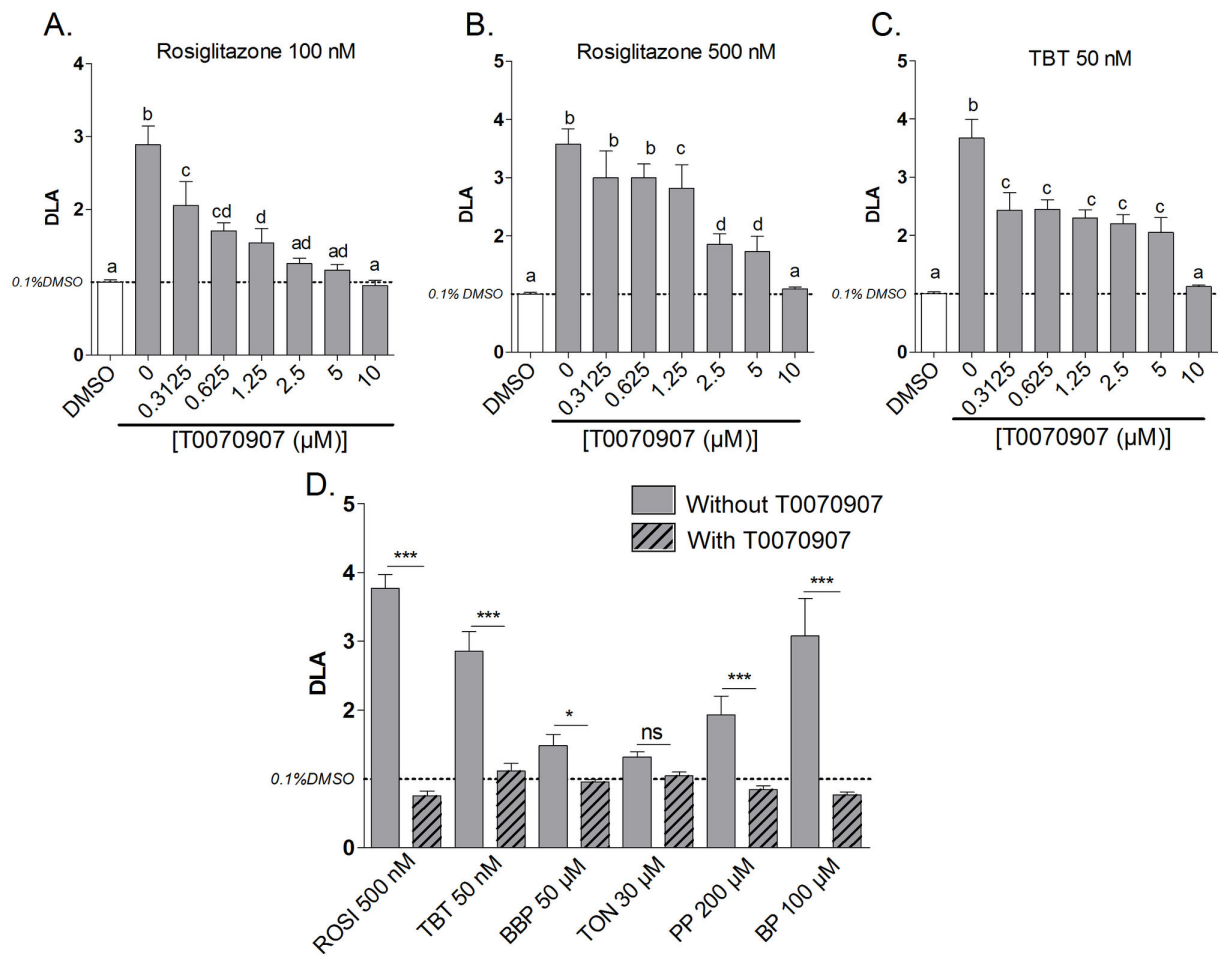
PPARγ dependency of the obesogenic effects of reference and test compounds. 3T3-L1 cells were exposed to Rosiglitazone 100 nM (A), 500 nM (B) TBT 50 nM (C) and screened obesogenic compounds (D) together with a concentration range of the PPARγ antagonist T0070907(A, B, C) or with and without 10 µM T0070907 (D). After 10 days of exposure fluorescent quantification of lipid accumulation was performed using Nile red staining. Data represent the degree of lipid accumulation (DLA) (mean ±SD) of 4 replicates. Significant differences (p≤0.05) between conditions are indicated with different letters for the optimisation tests (A, B, C) and significant differences between screened obesogens with and without T0070907 (D) are indicated with asterisks (*p≤0.05; **p≤0.01; ***p≤0.001) (One Way ANOVA Tukey’s post hoc test).

To evaluate the PPARγ dependence of the obesogenic effect of tested compounds, a PPARγ.antagonist assay was performed. The T0070907 antagonist test was first optimized by adding a concentration range of T0070907 to the known PPARγ agonists, ROSI (100 nM and 500 nM) and TBT (50 nM). A concentration dependent decrease of the DLA was observed and 10 µM of T0070907 was needed to fully suppress the TBT and ROSI mediated differentiation (Figure 8). This concentration was therefore selected for the next antagonist experiments. All strong obesogens screened by at least one selection criteria, with the 3T3-L1 adipocyte differentiation assay (DLA_MAX_>LAT or SSMD>4.7) were evaluated. Except for the musk compound TON, all effects were suppressed by adding 10µM of T0070907, showing the PPARγ dependency of parabens and BBP obesogenic action (Figure 8).

### PPARγ transactivation CALUX measurements

**Figure 9:**
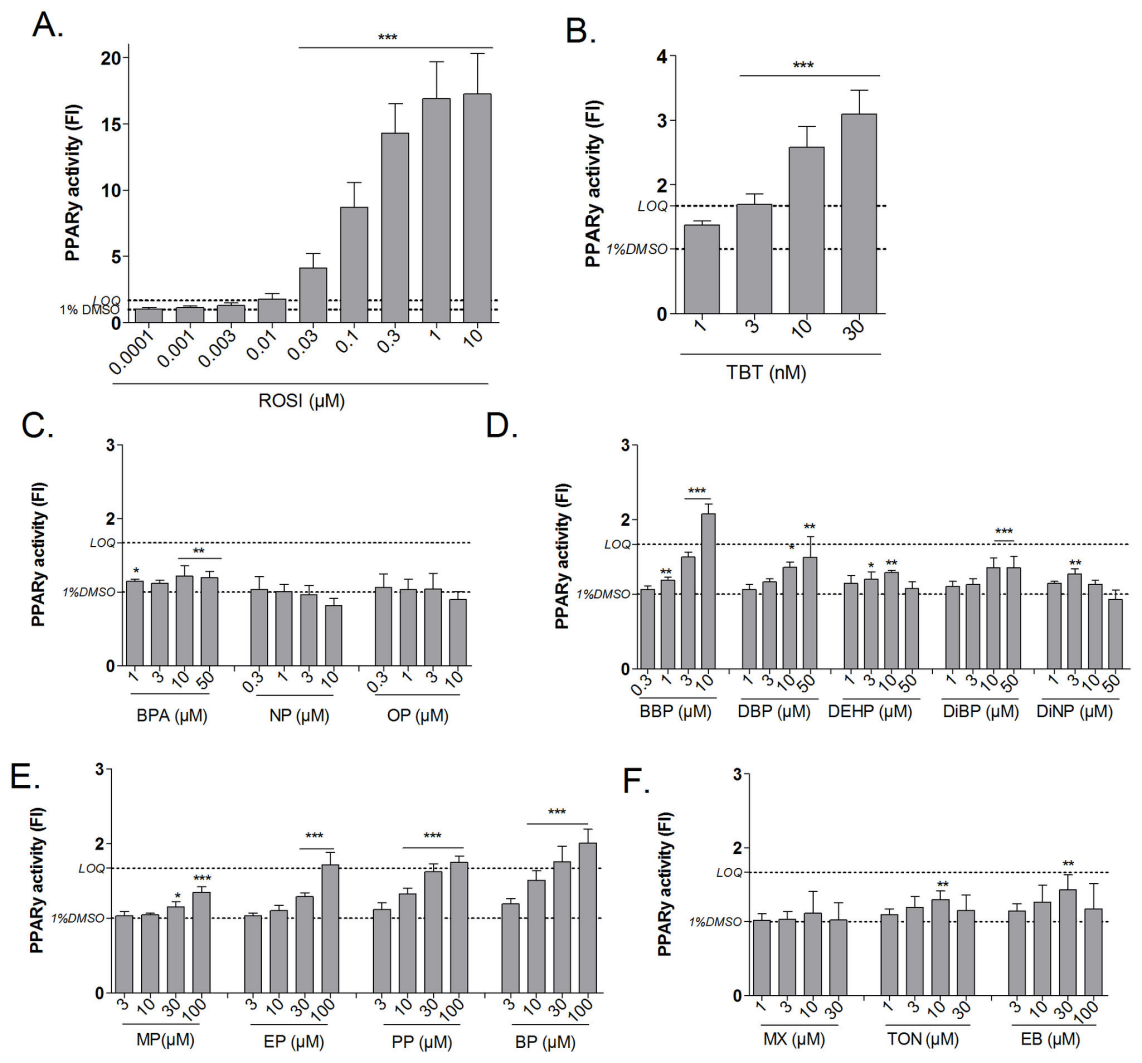
Effect of reference and test compounds on PPARγ activation in the PPARγ CALUX cell line. After 24h of exposure luciferase activity was measured with a luminometer. PPARγ activation of reference compounds ROSI (A.) and TBT (B.) and screened EDCs: Alkylphenol (C.), Phthalate (D.) Paraben (E.) and Musk (F) compounds are represented as mean (±SD) of the fold induction of at least three independent experiments performed in triplicate. Significant differences with solvent control (1% DMSO) are indicated with asterisks (One Way ANOVA Dunnet’s post hoc test; *p≤0.05; **p≤0.01; ***p≤0.001). *LOQ limit of quantification*.

In parallel to adipocyte differentiation, the compounds were tested on the PPARγ CALUX^®^ cell line to evaluate the PPARγ binding capacity of the tested compounds. For a standardization of the procedure, the fold induction (FI) of PPARγ activation was expressed as relative luminescence units (RLU) of the test compound treated cells relative to the RLU of the solvent control (1% DMSO) exposed cells. To ensure the sensitivity of the assay, ROSI was always included as positive control. Only experiments with a FI of ROSI treated cells greater than 10 were used. In concordance to the 3T3-L1 adipocyte differentiation assay, a LOQ value of 1.67 was calculated. Weak activators are defined as compounds that significantly activate the receptor for at least two subsequent concentrations, and strong activators induce a FI higher than the LOQ. This selection procedure was compared to the SSMD method, and compounds were classified as previously described for the adipocyte differentiation assay. Reference compounds, ROSI and TBT induced the activation of PPARγ dose-dependently (Figure 9A, B). The alkylphenol compound BPA was screened as a weak activator causing a FI_MAX_ of 1.22 (Figure 9C). Regarding the phthalates only BBP acted as a strong activator, whereas DBP, Bis(2-ethylhexyl)phthalate (DEHP) and DiBP acted as weak activators causing a FI_MAX_ of 2.08, 1.5, 1.28 and 1.35 respectively (Figure 9D). Concerning paraben compounds, they all activated the PPARγ receptor and as for the differentiation, the FI increased with increasing alkyl chain length (Figure 9E).PP and BP were strong activators inducing a FI_MAX_ of 1.75 and 2.01 respectively, whereas MP and EP were selected as weak obesogens inducing a FI_MAX_ of 1.35 and 1.71 respectively. The musk compounds showed no transactivation of the PPARγ receptor (Figure 9F). As for the differentiation experiments, dose response curves were constructed (graphs not shown) and based on the EC_LOQ_ values compounds were ranked from high to low potency giving the following order: TBT (2.9nM) > ROSI (9.9nM) > BBP (4.4µM) > BP (14.8µM) > PP (54.1µM).

## Discussion

The environmental obesogen hypothesis states that an exposure to environmental pollutants early in life or throughout life could influence the etiology of obesity [29]. Different modes of action of different obesogens have been proposed, some acting on the number or size of fat cells, other on hormones that affect appetite satiety or energy metabolism, but all resulting in an increased susceptibility for obesity development [30]. Different research groups already indicated the potential of organotins and other environmental pollutants to act as obesogens based on the potential to induce adipocyte differentiation [4]. However, no standardized protocol for testing the effect of compounds on adipocyte differentiation has been proposed. Previous studies measuring the adipogenicity of compounds measured the added effect of compounds after MDI induction of differentiation [6,7,31] or in combination with insulin [32]. Moreover, the time-points at which (varying from day 6 to day 10 after induction of differentiation), the method by which differentiation is measured and, the concentrations of the hormones present in the MDI cocktail differ from study to study. 

In this paper we propose to include the testing of single compounds and co-exposure with insulin to gather information on the adipocyte differentiation induction potential of the compound alone or when co-stimulated with insulin. In that way compounds that can trigger adipocyte differentiation, but are not able to convert triggered cells to mature adipocytes are picked up. Despite the fact that MDI triggered cells exposed to test compounds are physiologically relevant, the dynamic range for detecting compounds remains low due to the already strong induction by MDI. Therefore, this exposure scenario was not included in this study. 

### Evaluation of the 3T3-L1 adipocyte differentiation assay

Although adipocyte differentiation experiments are extensively been performed in the past using the 3T3-L1 cell line, the use of direct fluorescent measurement of Nile Red staining for detection of obesogens, has only scarcely been used. This despite the fact that this method has recently been presented as a highly quantitative and least subjective method compared to flow cytometric and Oil Red O photograph based quantification [18]. Therefore, in this paper a first step for standardisation of the 3T3-L1 adipocyte differentiation assay based on direct fluorescent measurement of Nile Red stained cells is proposed. 

In a first step, we thoroughly evaluated the 3T3-L1 adipocyte differentiation assay with two reference compounds: i) TBT as the most studied and evidenced environmental obesogen *in vitro*, *in utero* and *in vivo* [4,21] and ii) ROSI as a pure PPARγ agonist. ROSI is a pharmaceutical compound used for diabetic glycemic control, but inducing weight gain as an undesirable side-effect, and therefore provides a proof-of-principle for a PPARγ-induced obesogenic effect [33]. To compare the adipogenesis-inducing potential of different obesogenic compounds, two new terms were introduced to define the degree of adipocyte differentiation compared to solvent or insulin control respectively, namely degree of lipid accumulation (DLA) and degree of lipid accumulation with insulin (DLAI). Moreover, the effect was delimited by setting lipid accumulation threshold (LAT) values, defined as the lowest amount of differentiation that can be accurately quantified based on the variability of the cell system. Based on the variation of the control condition, an induction of approximately 2 times can be considered as sufficiently distinct from the control condition and thus representing a clear positive response. For the 0.1% DMSO solvent control, this threshold was substantially lower (1.76), than for the insulin control (2.17). Overall, an increased variability of the solvent control was seen in the insulin-compound co-exposure set up and can be attributed to the additional exposure step of these cells. The introduction of this threshold not only provides a critical evaluation of statistically significant responses, but was also used to discriminate weak from strong obesogenic compounds. Compounds inducing a higher DLA or DLAI than the LAT were considered as strong obesogens, whereas compounds inducing a DLA or DLAI lower than the LAT, but significantly different from the solvent control at two subsequent concentrations, were defined as weak obesogens. 

This new approach was compared to the SSMD method proposed by Zhang [27] for selection of hits in high throughput screening systems. The main differences between both approaches is that the LAT based approach is mainly based on the obesogenic induction potential of compounds, whereas using the SSMD value, the standard deviation of the effect is also taken into account. SSMD therefore seem advantageous in comparison to the LAT based approach. Nevertheless, when a compound induces a very small effect, with a small standard deviation it can be selected as a strong obesogen (e.g. MX in insulin-compound co-treatment exposure), whereas a compound inducing a high effect with a bigger standard deviation can then be missed (e.g. DBP in PPARγ CALUX). Therefore we think that combining both methods for a first line selection of obesogenic compounds is preferable. In this paper, the strongest obesogenic effect defined by either one of these methods was considered to classify the compounds. 

Experiments with reference compounds (TBT and ROSI) resulted in clear dose response relationships confirming the applicability of this setup for obesogen screening. Moreover the CV of the DLA_MAX_ values was very low (8.51% and 16,51% for respectively TBT and ROSI). Interestingly, the EC_LAT_ concentrations were lower for TBT compared to ROSI showing the potential of an environmental compound to act at even lower concentrations than a PPARγ agonistic pharmaceutical compound (Figure 6). 

For toxicity testing, comparison between the effects of a single compound with the effects of a single reference compound is preferable, as is the case for evaluating estrogenic or carcinogenic effects of chemicals. For these effects, reference compounds 17β-estradiol and benzo(a)pyrene (BaP) are respectively being used to calculate potency equivalence factors expressed as estradiol equivalent concentrations (EEC) or BaP equivalent concentrations [34,35]. Based on the results of this paper, we suggest using ROSI as reference compound to evaluate the adipogenicity of a compound based on the final lipid accumulation effects, based on the strong DLA(I) induction at low concentration, and the selection of ROSI as strong obesogen by both criteria (LAT and SSMD based). In that way, comparison of potency or effects of obesogenic compounds will be possible in the future. Using MDI as a reference treatment is too complex noting that this cocktail consists of 3 hormone compounds. Moreover, our results indicate that the response of MDI is less stable compared to the one-compound treatment such as ROSI response. 

Since PPARγ is a major regulator of adipocyte differentiation and a key mechanism for obesogen action [36], the potential PPARγ dependency of the obesogenic effect was evaluated with a PPARγ antagonists test. Furthermore, PPARγ activation was determined using a CALUX reporter cell assay. The PPARγ antagonist test was optimized using the known PPARγ agonists ROSI and TBT. Recently Li and colleagues [20] showed the instability of different PPARγ antagonists in cell culture, noting the importance of replenishing the media with the antagonist frequently and using ROSI as a positive control to ensure the effectiveness of the antagonist assay. Using our setup we were able to suppress the adipocyte differentiation induced by ROSI and TBT in a T0070907 dose-dependent manner. This confirmed the reliability of this assay to evaluate the PPARγ dependency of obesogenic compounds and indicated a strong involvement of PPARγ in the TBT-induced adipocyte differentiation effect *in vitro*.

### Screening of human-relevant environmental pollutants

To test the performance of this experimental setup, several classes of chemicals used in personal and household care products were tested, namely musks, phthalates, parabens and alkylphenol compounds. These groups of chemicals were selected because of the high exposure in daily life, through use of household products, personal care products, plastics, … making these chemicals compounds of great human interest [37]. Endocrine disrupting effects and in particular estrogenic effects of these compounds have previously been tested by others [12]. Moreover, the possible impact of compounds used in cosmetics on breast cancer development has been pointed out by different studies [38]. Besides these effects other health consequences due to a high chemical exposure (e.g. accumulation in the adipose tissue) cannot be ruled out. 

Musk compounds are a group of synthetic perfumes used in a variety of personal care products and can be divided into three different classes based on their structure: i) nitromusk, ii) polycyclic and iii) macrocyclic musk compounds [39]. The polycyclic musks, Tonalide (TON) and Galaxolide, represent about 95% of the musk market [39]. Moreover, an Italian study showed a detection of TON in 83% of the adipose samples tested and a 10 times increase in concentration of these musks during the last 20 years [17]. Nowadays, these musks are progressively being replaced by macrocyclic musk compounds considering their lower bioaccumulation potential [39]. During this study we evaluated the potential obesogenic properties of four musk compounds belonging to the different classes of musks: the nitromusk Musk xylene (MX); the polycyclic musk TON and the macrocyclic musk Ethylene Brassylate (EB). The lipid accumulation experiments listed TON as strong obesogen, whereas MX was screened as weak obesogen (Table 1). The LOEC is the highest for TON (7.5 µM) > MX (6.25 µM). When insulin is present the LOEC is lower for TON (0.94 µM) but not for MX (25 µM). Nevertheless, only MX was able to behave as a strong obesogen when insulin was added to the media. Moreover, the obesogenic effect of TON does not seem to be PPARγ mediated as shown by the PPARγ antagonist and activation test. One possible explanation for these results is a similar mechanism of action of TON and insulin, explaining why addition of insulin to TON does not change its induction potential. However, a more thorough mechanistic evaluation at the molecular level of the adipocyte differentiation is necessary before solid conclusions on obesogenicity can be drawn. 

Another group of tested compounds are the phthalates, widely used since the ’30 of previous century, in a variety of applications such as cosmetics, medicines, paints, medical equipment and most importantly in plastics (e.g. as plasticiser in PVC). Recently, prenatal exposure to Bis-(2-ethylhexyl) phthalate (DEHP) in mice was shown to induce obesity [40]. However, in our study, DEHP did not induce differentiation of 3T3-L1 cells. This indicates the possible limitations of the 3T3-L1 *in vitro* cell system lacking the metabolization of DEHP. Mono-ethyl-hexyl phthalate (MEHP) and mono-benzyl phthalate (MBzP), metabolites of respectively DEHP and butyl-benzylphthalate (BBzP), have been identified as PPARγ agonists and stimulate the differentiation of 3T3-L1 adipocytes [41-44]. Moreover, an epidemiological study showed a positive association between phthalate metabolite concentrations in human urine samples and BMI [43]. Other interesting phthalate compounds, due to their wide use in the EU, are Di-n-butylphthalate (DBP), butylbenzyl phthalate (BBP), di-iso-butyl phthalate (DiBP) of di-iso-nonyl phthalate (DiNP) [10]. DBP, BBP and DEHP were recently banned for use in toys in the EU mainly due to their reproductive toxicity. DiBP is now progressively being used as a substitute for DBP [45]. Our adipogenesis assay showed that BBP and DiBP were respectively strong and weak obesogens in the absence of insulin. However, based on LOECs, DiBP (25 µM) is more potent in comparison with BBP (50µM). Moreover, adding insulin to the media resulted in a strong effect of BBP and DiBP on adipocyte differentiation, with the same potency difference than in the single compound treatment (LOEC_BBP_=25µM; LOEC_DiBP_=12.5µM) (Table 1). A strong effect of DBP on adipocyte differentiation was observed in the insulin-compound co-treatment experiments. The CALUX binding assay indicated the weak activation of PPARγ by DBP, DEHP and DiBP and strong activation by BBP. Except for DEHP, these results are in concordance with our results from the 3T3-L1 adipocyte differentiation assay and with previous PPARγ reporter studies of others testing several EDCs [46]. Concerning DEHP, we are the first to show a PPARγ activation capacity [46]. However, it should be noted that the activation of PPARγ by DEHP is weak and therefore may not be strong enough to induce adipocyte differentiation. BBP was the only phthalate inducing a strong effect in the 3T3-L1 adipocyte differentiation and in the PPARγ CALUX assay. The PPARγ dependency of this effect was further confirmed by the suppression of the 3T3-L1 adipocyte differentiation after T0070907 addition. These results are in agreement with previous PPARγ reporter assays with phthalate compounds, where BBP was the only tested phthalate that was able to activate the nuclear receptor PPARγ [5,46]. To our knowledge, we are the first to describe a weak obesogenic activity of DiBP without insulin and an obesogenic effect of DiBP and DBP in the presence of insulin, and a weak PPARγ activation capacity of these phthalates.

Alkylphenol compounds are priority environmental contaminants and defined as EDCs on the *substances of very high concern* list in REACH. Their applications vary from anti-conception to use as non-ionic surfactants in a number of industrial, household and personal care products. Bisphenol A (BPA) has been shown to induce the differentiation of 3T3-L1 cells in combination with MDI treatment [6,47]. Additionally, neonatal BPA exposure in rats correlates with an increased bodyweight in the adult rats [32,47,48]. Our experiments confirmed this adipogenic potential, although it was screened as a weak obesogen regardless of the presence of insulin. Sargis et al. [7] have reported the glucocorticoid receptor (GR) binding capacity of BPA, indicating a possible GR mediated obesogenic mechanism of action. GR antagonist or silencing tests are however necessary to confirm this mechanism of action. BPA induced a weak activation of PPARγ using the CALUX assay, which is in concordance with Wang et al. [5] and indicates the possible dual mechanism of BPA through GR and PPARγ activation. Masuno et al. [49] and Masuno et al. [47] described the potential of Nonylphenol (NP) to induce the proliferation of mature adipocytes and inhibit the formation of mature adipocytes. These results are confirmed by our experiments showing a decrease in DLAI after NP exposure. Further in dept mechanistic studies are necessary to confirm this possible anti-obesogenic effect of NP. 

Parabens are used as preservatives in cosmetics due to their bactericide or fungicide properties. The chain length of the alkyl group is correlated with their antimicrobial activity and lipophilicity [50]. Our lipid accumulation results are in agreement with recent publications of Hu et al. [51] and Taxvig et al. [6] indicating the adipogenic potential of parabens and the increased adipogenic potential with increasing chain length. The PPARγ dependency of the effect is also confirmed by our PPARγ antagonist and receptor activation experiments. It should be noted that concentrations needed to induce adipogenic effects were high (50-200µM) compared to the concentrations measured in human tissues (10-80 nM) [12]. 

Considering *in vitro* testing, McKim [52] emphasized the importance to understand both the strengths and weaknesses of each model. It should therefore be noted that besides the advantage of being the most characterized cell system for adipogenesis, 3T3-L1 cells are pre-adipocytes, committed to the lineage of adipocytes. Therefore the study on early effects of commitment cannot be detected using this cell system. Therefore the promising multipotent stem cells (MSCs) could be a valuable alternative system [53]. However, previously described donor-to-donor variability and heterogeneous nature of MSCs might be a limitation of that cell system [54,55]. 

In conclusion, this paper proposes a standardized protocol to define the induction of adipocyte differentiation potential of compounds. With the introduction of terms such as DLA, DLAI and delimitating the effect using both LAT and SSMD values, comparison between different studies using the 3T3-L1 adipocyte differentiation assay will be possible. The results of this study indicate that this assay, based on direct fluorescent measurement of Nile red staining, can be considered robust, reproducible and sensitive to low concentrations (TBT and ROSI) and therefore interesting to be considered as a prioritization assay for further obesogen toxicity testing. Additionally, combination with the PPARγ-CALUX and the T0070907 PPARγ-antagonist assay is useful to have an indication of the PPARγ dependence of the obesogenic effect. Nevertheless, screening for PPARγ agonists as potential obesogens can be done using the PPARγ CALUX with the advantage that these tests can be performed quicker (2 days vs. 10 days). Furthermore, we suggest using ROSI as reference compounds for *in vitro* testing of obesogenic effects, which will allow comparing different studies using ROSI equivalent concentrations. Moreover, we confirm the obesogenic properties of parabens, BPA and some of the tested phthalates. Interestingly, we are the first to show the adipogenic potential of musk compounds, suggesting a non-PPARγ mediated mechanism and a PPARγ mediated obesogenic effect of the phthalates DiBP and DBP. Nevertheless, it should be noted that the concentrations at which the screened compounds induced the obesogenic effect (µM range) is higher than that of the reference compounds (nM range). This study, however, only gives a first indication of the potency of compounds to interact with key mechanisms in energy metabolism. Further in-depth *in vitro* and *in vivo* research will be crucial to fully comprehend their mechanistic profiles and to fully understand their potential role in the aetiology of obesity.
